# Identification of new potent NLRP3 inhibitors by multi-level in-silico approaches

**DOI:** 10.1186/s13065-024-01178-3

**Published:** 2024-04-18

**Authors:** Chandni Hayat, Vetriselvan Subramaniyan, Mubarak A. Alamri, Ling Shing Wong, Asaad Khalid, Ashraf N. Abdalla, Sahib Gul Afridi, Vinoth Kumarasamy, Abdul Wadood

**Affiliations:** 1 Department of Biochemistry, Abdul Wali Khan University, Mardan, Mardan, 23200 Pakistan; 2 Pharmacology Unit, Jeffrey Cheah School of Medicine and Health Sciences, Monash University, Malaysia, Jalan Lagoon Selatan, Bandar Sunway, 47500 Subang Jaya, Selangor Darul Ehsan Malaysia; 3 Department of Pharmaceutical Chemistry, College of Pharmacy, Prince Sattam Bin Abdulaziz University, 11942 Al-Kharj, Saudi Arabia; 4 Faculty of Health and Life Sciences, INTI International University, 71800 Nilai, Malaysia; 5 Substance Abuse and Toxicology Research Center, Jazan University, P.O. Box: 114, 45142 Jazan, Saudi Arabia; 6 Department of Pharmacology and Toxicology, College of Pharmacy, Umm Al-Qura University, 21955 Makkah, Saudi Arabia; 7 Department of Parasitology and Medical Entomology, Faculty of Medicine, Universiti Kebangsaan Malaysia, Jalan Yaacob Latif, 56000 Cheras, Kuala Lumpur Malaysia; 8 Center for Global Health Research, Saveetha Medical College, Saveetha Institute of Medical and Technical Sciences, Chennai, 602105 India

**Keywords:** NLRP3, Diabetes mellitus, Inflammasome, Inhibitors, Pharmacophore model

## Abstract

Nod-like receptor protein 3 (NLRP-3), is an intracellular sensor that is involved in inflammasome activation, and the aberrant expression of NLRP3 is responsible for diabetes mellitus, its complications, and many other inflammatory diseases. NLRP3 is considered a promising drug target for novel drug design. Here, a pharmacophore model was generated from the most potent inhibitor, and its validation was performed by the Gunner-Henry scoring method. The validated pharmacophore was used to screen selected compounds databases. As a result, 646 compounds were mapped on the pharmacophore model. After applying Lipinski's rule of five, 391 hits were obtained. All the hits were docked into the binding pocket of target protein. Based on docking scores and interactions with binding site residues, six compounds were selected potential hits. To check the stability of these compounds, 100 ns molecular dynamic (MD) simulations were performed. The RMSD, RMSF, DCCM and hydrogen bond analysis showed that all the six compounds formed stable complex with NLRP3. The binding free energy with the MM-PBSA approach suggested that electrostatic force, and van der Waals interactions, played a significant role in the binding pattern of these compounds. Thus, the outcomes of the current study could provide insights into the identification of new potential NLRP3 inflammasome inhibitors against diabetes and its related disorders.

## Introduction

The immune system defends against external and internal pathogens by using innate and adaptive immunity [1]. The innate immune system is the body’s first line of defense against foreign invaders. Various pattern recognition receptors (PRRs) are used by the innate immune system to determine consistent microbial patterns [2]. NOD-like receptors (NLRs) are described as intracellular PRRs which play an essential role in sensing the molecules linked with intracellular receptors and stress conditions. Thus, they sense different stimuli which present microbial infection and damage [3]. The NLRs consist of various members, including NLRP2, NLRP3, NLRP4, NLRP6, NLRP7, NLRP10, and NLRP12 [4]. Among them, NLRP3 is the most characterized member of the NLRs family. It is organized into three domains: an amino-terminal pyrin domain (PYD), a central nucleotide-binding domain (NACHT), which exhibits ATPase activity, also promotes self-oligomerization and a carboxy-terminal leucine-rich repeat (LRR) domain that acts as auto-inhibitory domain and plays an active role in signaling [5]. The PYD, NACHT, and LRR domains containing protein-3 were discovered as an important part of inflammasome [6, 7]. The inflammasomes are multi-protein complexes, composed of sensor protein (NLRP3), adaptor protein apoptosis-associated speck-like protein (ASC), and pro caspase-1 [8]. Under favorable conditions, the NLRP3 inflammasome localized at cytosol. Meanwhile, the presence of exogenous invaders or activators such as pathogens-associated molecular patterns, damage-associated molecular patterns, and environmental stress allows NLRP3 to associate with ASC by homotypic PYD interactions. Then, ASC binds with pro caspase-1 via homotypic caspase recruitment domain (CARD), and forming NLRP3 inflammasome complex. The caspase-1 facilitates the maturation of cytokines, (IL)-1β and interleukin-18, which starts the inflammation process, termed as pyroptosis [9–11]. Aberrant expression of NLRP3 contributes to multiple inflammatory disorders, including obesity, diabetes, dyslipidemia, hypertension, traumatic brain injury, and cerebrovascular disease [12–17]. Accumulating evidence shows the relationship between diabetes and NLRP3 inflammasome. Tschopp et al. first reported that NLRP3 act as sensor for metabolic danger that might facilitate the diabetes progression [18]. NLRP3 inflammasome activation affects insulin sensitivity and glucose tolerance, which promotes (IL)-1β and interleukin-18. The over expression of (IL)-1β causes endoplasmic reticulum stress and oxidative stress, which can lead to pancreatic cell death, affects T-cell activation, disrupt the function of islets β cells, and lead to diabetes [19]. A recent study reported that NLRP3 over expression contributes to diabetes related disorders, including atherosclerosis, diabetic nephropathy, and cardiomyopathy. Despite the advancement in therapeutic options, the management of diabetes and its complicated disorders become a significant challenge. The development of novel inhibitors by targeting the NLRP3 inflammasome, is a key player in pathogenesis of these disorders. Recently, computer aided drug discovery changed the way in which most effective drugs are designed by allowing the researchers to screen, and analyze the large database of compounds for therapeutic candidates against targeted proteins [20]. In this study, we used different computational approaches, including Pharmacophore-based virtual screening, molecular docking, MD simulations, and MM-PBSA calculations to predict the new and potential inhibitors against NLRP3 protein.

### Computational methodology

#### Ligand-base pharmacophore generation

A pharmacophore is a chemical framework which contains the structure features of known active compounds. A ligand based pharmacophore model is developed based on known active, and specific compound, Tranilast [21] by using the pharmacophore query editor wizard of molecular operating environment (MOE). The MOE used an in-built set of pharmacophore features containing an aromatic center, H-bond donor, H-bond acceptor, cationic or anionic atom, hydrophobic atom, and Pi ring center [22]. In current study, the essential chemical features i.e. hydrogen bond donors, hydrogen bond acceptors, atom Q, and hydrophobicity were utilized to develop pharmacophore model.

#### Pharmacophore validation

Analysis of validated pharmacophore is essential to ensure the accuracy of molecular model and verify its reliability [23]. The developed pharmacophore model was validated by the Guner-Henry (GH) method [24], in which the internal database comprised of in-active (1319) (decoys) and experimentally active (150) compounds. The active inhibitors for NLRP3 were retrieved from literature and decoys were obtained in smile format from active inhibitors by LiDEB server, and builder in MOE was used for the generation of three dimensional structures [25], and the internal database screening was carried out through pharmacophore search protocol in MOE.

GH score was calculated by a mathematical formula.

1 $${\text{GH}} = \left[ {{\text{Ha}}\left( {{\text{3A}} + {\text{Ht}}} \right)/{\text{4HtA}}} \right] * \left( {{1} - {\text{Ht}} - {\text{Ha}}/{\text{D}} - {\text{A}}} \right)$$

The statistical parameter of Ht presents a number of compounds considered as hits; Ha indicates a number of actual actives in hits; A express number of active molecules in internal database; D indicates total number of molecules in internal database. The higher score of GH represents the performance of pharmacophore model is good. The Pharmacophore model was further used for virtual screening process.

##### Evaluation of drug like features

The pharmacophore-based virtual screening of databases are considered to be the most crucial tool for drug discovery and brings information about electronic and geometric features that are involved in binding interaction with receptor [26]. Prior to conducting the virtual screening, ZINC (12,000 molecules), In-house (1700 molecules), and Phytochemical (5000 molecules) databases were used. These ZINC, and Phytochemical databases are the largest and openly available databases, usually employed to identify the most effective inhibitors against different diseases. To generate more refined and precise databases, all the databases were cleaned, subjected to 3D protonation by using the MMFF94 force field and energy minimized with an RMS gradient of 0.05 by using the energy minimize application in MOE [27]. In addition, the force field partial charges were computed and hydrogen was added. Because of screening 349, 186, and 111 hits were extracted from ZINC, In-house, and Phytochemical libraries, respectively. Identification of drug-likeness predicts new hits by virtual screening [28]. Therefore, the screened compounds were filtered by Lipinski rule of five (RO5), and ADMET [29, 30], for searching the drug-likeness properties and pharmacokinetics, respectively. The screened compounds were short-listed by RO5 in MOE. According to RO5, a drug like molecule has a hydrogen bond donors (< 5), hydrogen bond acceptors (< 10), molecular weight (< 500 Da), polar surface area not greater than 140 Å and lipophilicity (logP < 5). Pan assay interference compounds (PAINS) (http://biosig.unimelb.edu.au/pkcsm/predication) is an electronic filter used to study the compounds quality in the database. PAINS analyze the compounds which are chemically reactive, more assay interfering, predict pharmacokinetics properties of screened compounds. Thus, it is important to study the combination of filtered compounds in order to obtain the desired pharmacokinetics properties. For the analysis, the smile format of the compounds were passed through filter in PAINS server and ADMET properties were predicted. All the compounds that obeyed the RO5 and passed the PAIN filters were subjected to molecular docking and MS simulation studies.

#### Molecular docking

Molecular docking analysis predicts the binding interaction between macromolecules or small molecules with protein receptors at the atomic level. It can identify the binding mode of small molecules into the protein-binding site [31–34].

#### Structure preparation

The three-dimensional structure of NLRP3 with (PDB ID: 7ALV) was retrieved in PDB format from RCSB Protein Data Bank. Before molecular docking, protein structure was prepared, correcting the protein structural issues, structure protonation, and structure minimized to a specific gradients, by using the quick prep option of MOE. Water molecules and other co-factors from protein structure were eliminated and H-atoms were added [35].

#### Active site residues prediction

For molecular docking study, active sites was selected from the literature. The active site residues of NLRP3 such as Ala226, Ala227, Arg351, Pro352, Arg578, and lys232, reported by ishania and dekker were used for molecular docking study [35, 36]. In the docking parameter of MOE, we set force field to MMFF94x, obtaining the gradient setting of 0.05 kcal/mol and the triangle matcher placement algorithm was applied. London dG method was used to score the poses [37]. After examining the docking results, total 6 hits from these databases were selected for MD simulations based on binding interaction, and S score. The S score observed the interactions, the lower S score with inhibitors interacting strongly with NLRP3 protein [38].

#### Molecular dynamic (MD) simulation

Combining the docking results with MD simulations allows the validation of docking results by confirming the conformational flexibility and structure stability of protein–ligand complexes. Therefore, the protein–ligand complexes were used to interact in a simulated environment for a specific period and trajectories were computed, affording the overall data by molecular motions as a function of time. MD simulation was employed via Amber2022 [39]. Force field plays significant role in MD simulation as they compute the potential energy of protein–ligand complexes [40]. In this work, generic AMBER force field (GAFF) was used for ligands and the FF14SB AMBER force field was applied for protein. Both topology and coordinates files for each system were built by tleap module of AMBER 2022 software, however, the atomic charges and topology file for ligands were built by antechamber suite in AMBER 2022 [41]. Solvation is essential as it enables studying the internal motion of protein at different temperature. A truncated octahedral box of TIP3P molecules of water was used for solvating the system and water molecules were added with LEap module of Amber2022. An appropriate amount of Na^+^ ions were added to neutralize the system. The Particle Mesh Ewald (PME) step was then employed to compute long range electrostatic and a cut-off distance was adjusted to 10 Å for the non-bonded interactions [42]. The SHAKE algorithm was monitored to constrain hydrogen containing bonds, after that, each system was heated from 0 to 300 K, and then and then equilibration was carried out at constant pressure and 300 K temperature. Finally, 100 ns production was completed of top six complexes along with reference compound [43, 44].

#### Post dynamic analysis

The generated trajectories from MD simulation of NLRP3 and hits were saved. Post MD analysis was performed by implementing the CPPTRAJ module in AMBER2022 package.

#### MMPBSA binding free energy

The binding free energy of protein–ligand complexes was calculated to signify their thermodynamic stability and binding affinity, which are closely associated with compound’s potency [45]. In present study, the Poisson–Boltzman (MM-PBSA) technique was used to compute the binding energy of all complexes.

The binding free energy was calculated by following equations.

$$\Delta G \, ({\text{bind}}) \, = \Delta G \, ({\text{complex}}) - \, \left[ {\Delta G \, ({\text{receptor}}} \right) \, + \, \Delta G\,({\text{ligand}})]$$

where ΔG bind represents binding free energy; ΔG complex denotes free energy of complex; ΔG ligand and ΔG receptor are free energy of ligand and receptor in complex system, respectively.

#### Dynamical cross-correlation matrix (DCCM)

DCCM were generated to better understand protein dynamics by analyzing cross correlation shift of backbone atoms. DCCM helps to provide protein dynamic simulation, representing how atomic displacement is coupled [46–48], denote amino acids correlation. In order to determine DCCM, the following equation was used.

$$C_{ij } = \frac{{\left\langle {\Delta {\text{r}}_{{\text{i}}} \cdot } \right.\left. {\Delta {\text{r}}_{{\text{j}}} } \right\rangle }}{{\left( {\left\langle {\left. {\left. {\Delta {\text{r}}_{{\text{I}}} } \right\rangle } \right\rangle } \right.^{{\mathbf{2}}} < \, \Delta {\text{r}}_{{\text{J}}} < \Delta {\text{r}}_{{\text{i}}} >^{{\mathbf{2}}} } \right){1 \mathord{\left/ {\vphantom {1 2}} \right. \kern-0pt} 2} \, }}$$

Both, Δr_i_ and Δr_j_ were displacements from mean position of i^th^ and j^th^ atoms respectively. The resulting values computed were from (− 1 to 1). A negative value represents negatively correlated movement and a positive value implies positive correlated motion [49].

## Result

### Ligand base pharmacophore model generation and validation

The pharmacophore was generated by using known active compound, Tranilast with (Pub Chem ID 5282230). Then, internal database was generated, which is composed of 150 active compounds and 1318 inactive compounds to properly assess the discriminative ability of the generated pharmacophore model by GH method. The generated database underwent screening against the pharmacophore model to verify its accuracy. The model identified 8 compounds, of which six were active and two were inactive, which indicates that model can differentiate between active and inactive compounds. Several essential parameters were calculated including active hits (Ha), total hits (Ht), % ratio of actives, % yield of actives, Enrichment factor (E), and GH score which were illustrated in Table 1. GH score between 0.7 and 0.8 presents a good model [50]. The goodness score of our validated pharmacophore was 0.76, which indicated that our resultant pharmacophore was efficient to be further used against different databases for virtual screening purpose.

**Table 1:** Validation of pharmacophore model by GH score

NO	Parameters	Model assessment
1	Total molecules in database (*D*)	1468
2	Total number of actives in database (*A*)	150
3	Total hits (*Ht*)	8
4	Active hits (*Ha*)	6
5	% Yield of actives[(*Ha*/*Ht*) × 100]	75
6	% Ratio of actives [(*Ha*/*A*) × 100]	4
7	Enrichment factor (E) [(Ha × D)/(Ht × A)	7
8	False negatives [*A*−*Ha*]	144
9	False positives [*Ht*−*Ha*]	2
10	Goodness of hit score (*GH*)	0.76

**Fig. 1:**
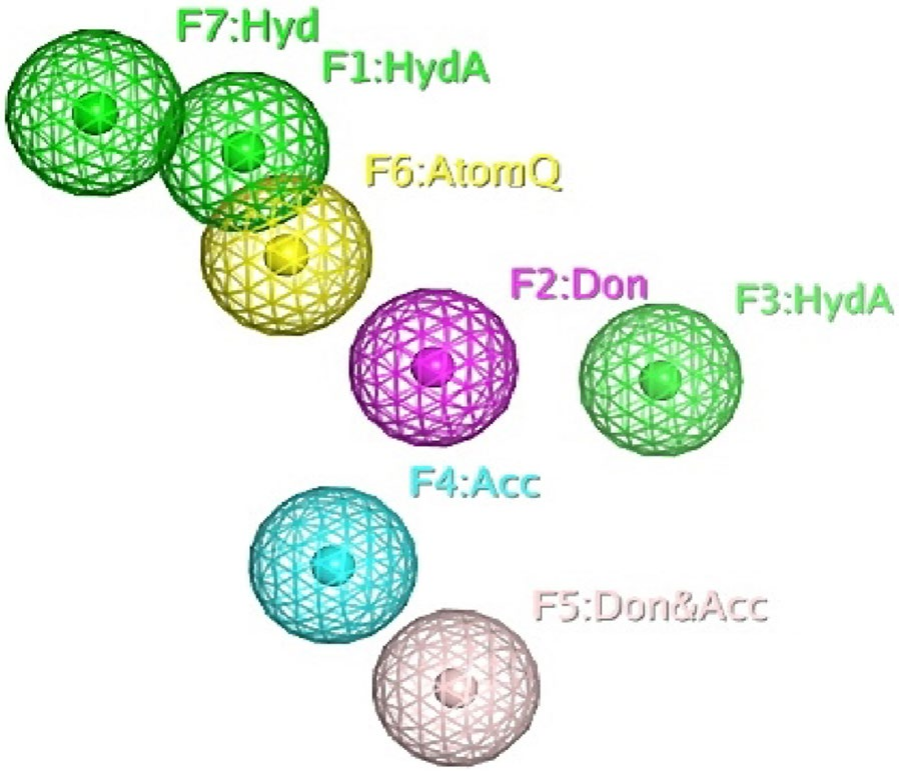
Chemical characterization of reference compound, F1: HydrA (green), F2: H-bond donor (purple). F3; HydrA (green), F4; H-bond Acceptor (blue), F5; H-bond donor and Acceptor (light purple), F6; Atom Q (yellow), F7; Hydrophobic (green)

The generated pharmacophore model has seven pharmacophore features, including two Hydrophobic Atom (F1 and F3), one H-bond donor (F2), one H-bond acceptor (F4), one H-bond donor and acceptor (F5), Atom Q (F6) and one Hydrophobic (F7) as illustrated in (Fig. 1).

### Virtual screening

The validated pharmacophore model was used to screen ZINC, In-house, and Phytochemical databases to identify new hits. Subsequently, because of screening, 349, 186, and 111 structurally diverse hits from ZINC, In-house, and Phytochemical databases were retrieved, respectively. Compounds that obey Lipinski’s rule of five are considered to be active in the human body [51]. Therefore, our study examined various properties including H-bond donor, molecular weight, logP value, and H-bond acceptor of compounds. As a result of Lipinski's rule of five filtering, 205, 101, and 85 hits that meet the criteria for drug-like characteristics were obtained for ZINC, In-house, and Phytochemical databases, respectively.

### Molecular docking analysis

In the MOE (2016) software, molecular docking was carried out to explore the binding interaction of ligands against target protein. We docked 205, 101 and 85 diverse hits from ZINC, In-house and Phytochemical databases, respectively, into active sites of NLRP3 NACHT domain. The docking results from MOE revealed that hits could be strongly accommodated into the binding pocket of NLRP3. From each database, the top 50 ranked conformations of protein–ligand complexes were saved based on docking score. The resultant binding interaction between protein and hits were visualized. We identified 30 best hits after filtering those inhibitors, which possess interactions with active site residues.

### Hits optimization on the basis of binding affinity and binding energy

**Fig. 2:**
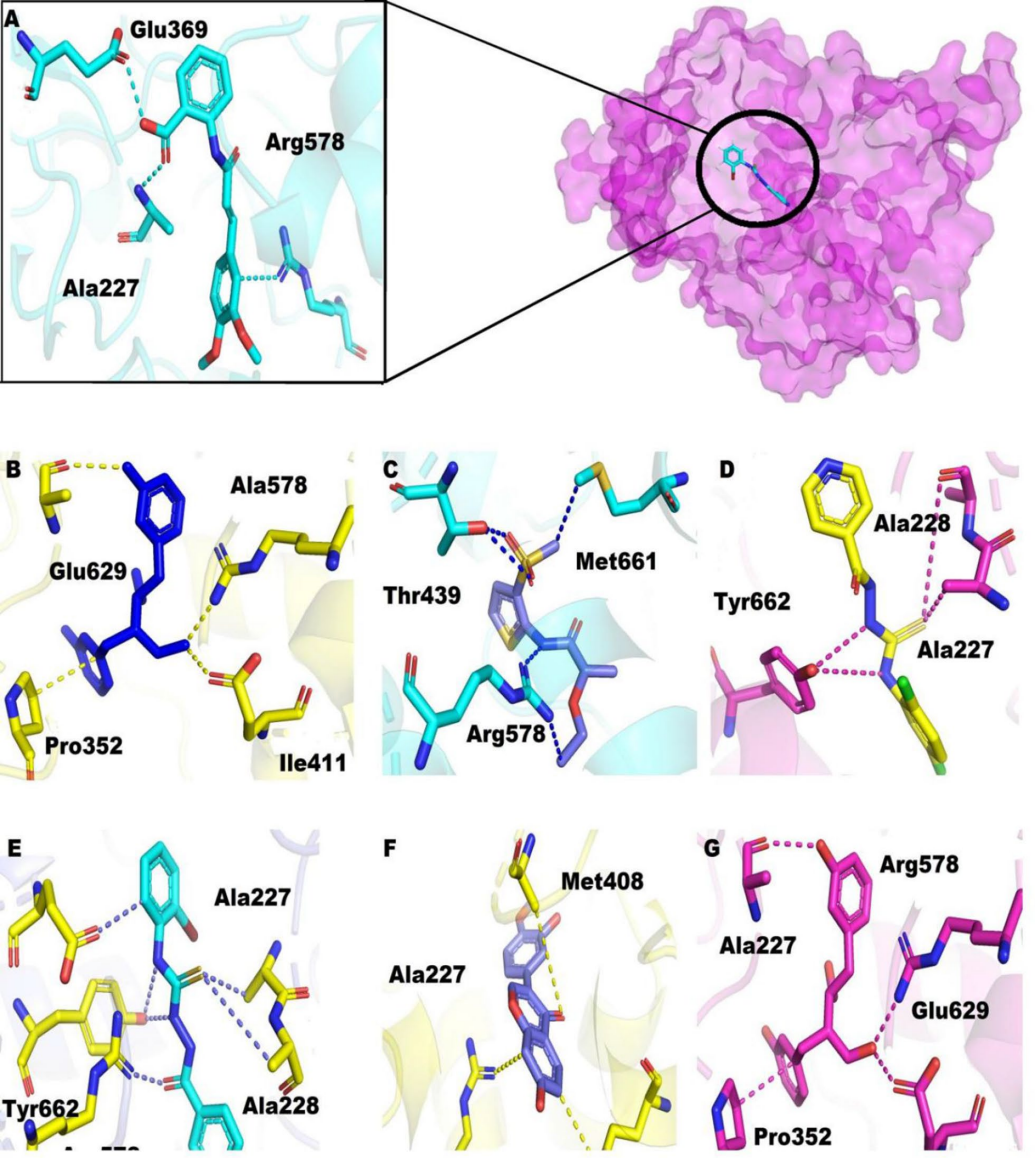
Binding interaction of protein–ligand; **A** Interaction of Tranilast; **B** Ligand interaction of ZINC12359085; **C** Ligand interaction of ZINC72288245; **D** Ligand interaction of BA-II-51; **E**. Ligand interaction of BA-II-45; **F** Ligand interaction of 5,280,448; **G** Ligand interaction of 115,089

The binding interaction and binding energy of selected inhibitors (30) from each library along with reference compound were studied. Finally, the 06 best hits (02 from each database) were finalized based on docking score and interaction (Table 2). As demonstrated in (Fig. 2a), the binding mode of reference compound in the active site of NLRP3 showed two hydrogen bonds with active site residues of NLRP3 (Ala227, Glu369) and one pi-cation (Arg578) along with binding score − 8.456 kcal/mol (Table 2). In ZINC database, compound ZINC12359085 exhibited one pi-H bond with Ile141 and four H-bonds with Pro352, Arg578, and Glu629 (Fig. 2b), with − 8.435 kcal/mol docking score. Similarly, compound ZINC72288245 demonstrated five hydrogen bonds (Thr439, Thr439, Arg578, Arg578 and Met661) in the binding site of NLRP3 (Fig. 2c). The compound ZINC72288245 revealed − 9.897 kcal/mol docking score.

**Table 2:** Protein ligand interaction (PLI) detail of 06 top most compounds in complex with NLRP3 NACHT domain

Compound	Docking score	Docking interaction result							
		Ligand		Receptor		Interaction		Distance E	E(kcal/mol)
ZINC12359085	− 8.435	S1	8	OE1	GLU	629	H-donor	3.57	− 2.5
		N2	10	CB	PRO	352	H-acceptor	3.34	− 0.6
		03	13	NH1	ARG	578	H-acceptor	2.78	− 3.2
		03	13	NH2	ARG	578	H-acceptor	2.97	− 2.0
		6-ring		CD1	ILE	411	Pi-E	3.61	− 0.6
ZINC72288245	− 9.897	C10	17	SD	Met	661	H-donor	4.09	− 0.6
		O1	3	NH2	ARG	578	H-acceptor	3.52	− 1.3
		03	15	OG1	THR	439	H-acceptor	2.78	− 2.2
		04	16	OG1	THR	439	H-acceptor	2.79	− 0.8
		04	16	NH1	ARG	578	H-acceptor	3.36	− 1.8
BA-II-51	− 12.654	S	12	CA	ALA	227	H-acceptor	3.92	− 0.5
		S	12	N	ALA	228	H-acceptor	4.31	− 1.8
		N	9	6-ring	THR	662	H-pi	4.30	− 0.9
		N	13	6-ring	THR	662	H-pi	4.45	− 0.9
BA-II-45	− 10.987	C	15	OE1	GLU	629	H-donor	3.36	− 0.5
		0	11	NH1	ARG	578	H-acceptor	2.67	− 0.7
		S	12	CA	ALA	227	H-acceptor	3.77	− 0.5
		S	12	N	ALA	228	H-acceptor	3.43	− 2.5
		N	9	6-ring	THR	662	H-pi	4.33	− 1.0
		N	13	6-ring	THR	662	H-pi	4.27	− 0.9
5,280,448	− 7.987	0	4	CA	ALA	227	H-acceptor	2.90	− 0.5
		0	1	NH2	ARG	578	H-acceptor	2.97	− 0.9
		6-ring		N	MET	408	H-donor	3.70	− 0.9
115,089	− 9.478	CO	1	OE1	GLU	629	H-donor	3.03	− 1.7
		0	4	0	ALA	227	H-donor	2.90	− 0.5
		0	1	NH2	ARG	578	H-acceptor	2.97	− 0.9
		6-ring		CG	PRO	352	Pi-H	3.70	− 0.9
Tranilast (Reference compound)	− 8.456	0	15	OE1	GLU	369	H-donor	2.84	− 2.2
		0	11	N	ALA	227	H-acceptor	3.21	− 0.8
		6-ring		NH1	ARG	578	Pi-cation	3.71	− 0.5

In case of In-house database, compound BA-II-51 formed two H-bonds (Ala227, Ala228) and two pi-H bonds (Thr662) with binding pocket residues along with a docking score of − 12.654 kcal/mol (Fig. 2d**)**. In addition, compound BA-II-45 exhibited four hydrogen bonds with various residues (Ala227, Ala228, Arg578, and Glu629) and two pi-H bonds with Thr662. For this compound, the docking score of − 10.987 kcal/mol was observed (Fig. 2e).

As depicted in Fig. 2f, compound 5,280,448, formed three hydrogen bonds with Ala227, Met408, Arg578 residues and − 7.987 kcal/mol docking score. Similarly, compound 115,089, formed three H-bonds (Ala227, Arg578, and Glu629) and pi-H bond interactions (Pro352) with − 9.478 kcal/mol docking score Fig. 2g.

The detail of binding interactions of the top 6 hits along with reference compound are presented in Fig. 2.

### PAINS filter assay

During drug designing, it is important to pass the compounds into various filtration for desirable pharmacokinetics properties. Therefore, all the six compounds along with reference were successfully passed through electronic filter. The results indicate that these compounds have desirable pharmacokinetics properties. Table 3 presented the PAINS filter results, compound structures and their IUPAC names.

**Table 3:** PAINS results of selected compounds along with their structures and IUPAC names

Ligand	PAINS filter	Structure	IUPAC name
ZINC12359085	Passed		5-((phenoxycarbonyl)amino)-1,2,3-thiadiazol-4-yl propionate
ZINC72288245	Passed		2-ethoxy-n-(3-(methylsulfonyl)thiophen-2-yl)propanamide
BA-II-51	Passed		n-(2,5-dichlorophenyl)-2-isonicotinoyl-2-methylhydrazine-1-carbothioamide
BA-II-45	Passed		n-(2-bromophenyl)-2-nicotinoylhydrazine-1-carbothioamide
5,280,448	Passed		7-hydroxy-2-(4-hydroxy-3-methoxyphenyl)-4H-chromen-4-one
115,089	Passed		2,3-bis(3-hydroxybenzyl)butane-1,4-diol
Reference compound	Passed		2-(3-(3,4-dimethoxyphenyl)propanamido)benzoic acid

### Molecular dynamic (MD) simulation and its analysis

The MD simulation was carried out to check the stability of selected compounds along with reference compound. The trajectories analysis was performed by using the CPPTRAJ module of AMBER software and post simulation analysis such as RMSD, RMSF, DCCM, PCA, hydrogen bond analysis, and binding free energy calculation were performed.

### Root mean square deviation

Root mean square deviation evaluates the differences in the backbone of protein complexes, from its initial structural state to final conformation state [52].

**Fig. 3:**
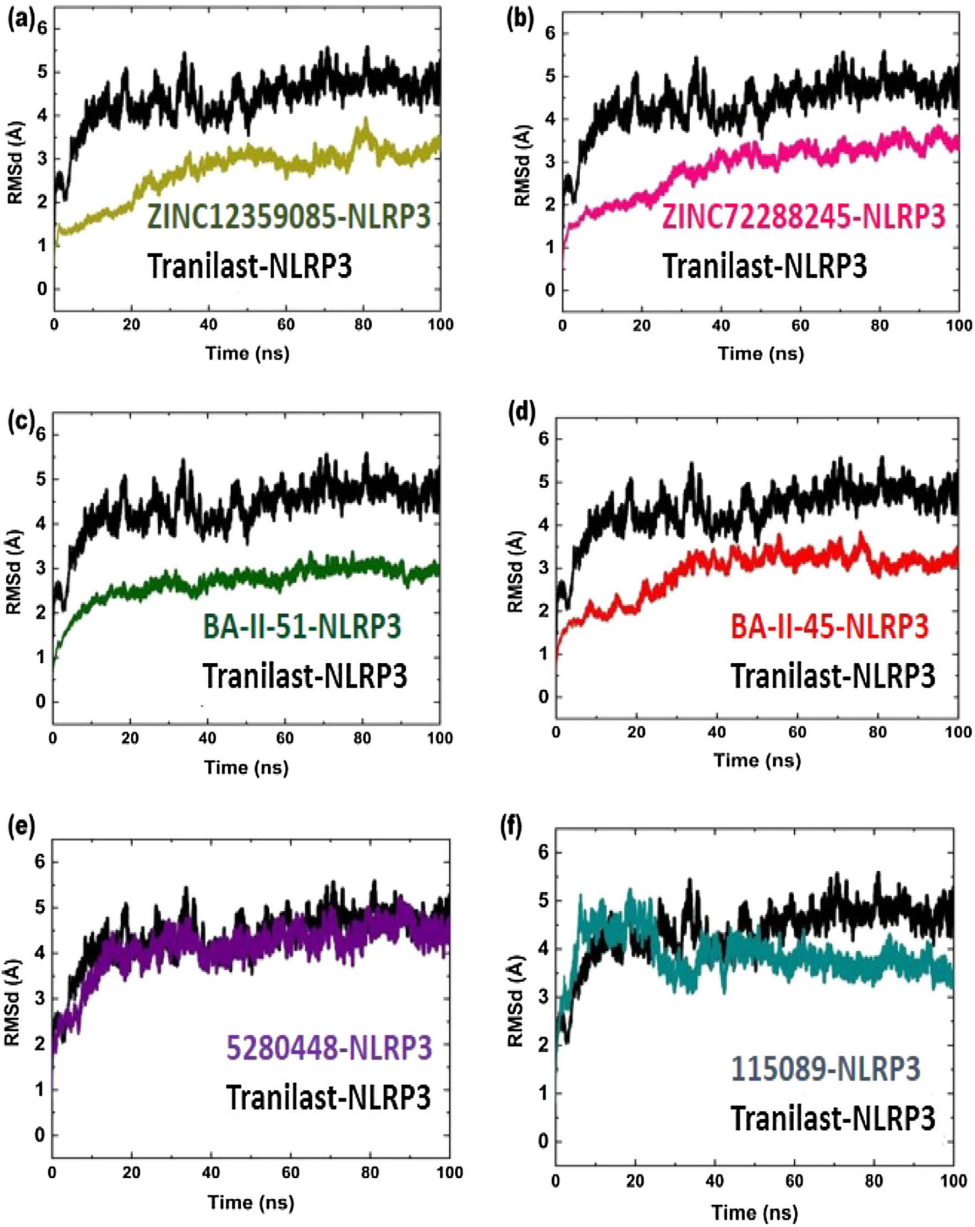
Black color in all graphs presenting RMSD for Tranilast-NLRP3 complex **a** ZINC12359085-NLRP3 complex **b** ZINC2288245-NLRP3 complex **c** BA-II-51-NLRP3 complex **d** BA-II-45-NLRP3 complex **e** 5,280,448-NLRP3 complex **f** 115,089-NLRP3 complex

The larger deviation curve represents lower stability and smaller deviation curve represents higher stability. The MD simulation results revealed that reference compound showed high deviation as compared with other selected compounds (Fig. 3). The RMSD curve for Tranilast-NLRP3 complex presenting 5–5.5 Å deviation throughout MD simulation period, presents maximum instability.

In ZINC database, the ZINC12359085-NLRP3 complex showed low RMSD value of 1.5–3.0 Å during 70 ns simulation period. Moreover, it oscillates at 3.5 Å around 75–85 ns, afterward gradually decreased (Fig. 3a). The ZINC72288245-NLRP3 complex revealed low deviations during 30 ns, after that it drastically jumped to 3.2 Å from 45 to 90 ns during MD simulation period (Fig. 3b). However, the complexes were more stable than reference compound.

The dynamic stability of BA-II-51 and BA-II-45 were computed to identify the binding stability. Interestingly, BA-II-51-NLRP3 complex presented local deviation, (1.2–2.5 Å), showed a dynamically stable behavior than other selected compounds (Fig. 3c). Therefore, our results showed that the BA-II-51-NLRP3 complex was more stable than Tranilast-NLRP3 complex, as it presented little deviation and more stable behavior than reference compound. As shown in (Fig. 3d), the RMSD curve for BA-II-45-NLRP3 complex showed 1–2.5 Å around 30 ns, but later jumped to 3.2–3.5 Å, during 30-80 ns simulation period. On the other hand, in phytochemical database, the RMSD graph for 5,280,448-NLRP3 complex exhibited 3.5-5 Å during MD simulation, showed unstable behavior like reference compound (Fig. 3e). In addition, the 115,089-NLRP3 complex exhibited high fluctuations 10–30 ns at 5 Å RMSD, which presented comparatively unstable behavior unlike other compounds. (Fig. 3f). We also calculated an average RMSD curve for complex systems. The average RMDS values for ZINC12359085-NLRP3, ZINC72288245-NLRP3, BA-II-51-NLRP3, BA-II-45-NLRP3, 5,280,448-NLRP3, 115,089-NLRP3, and Tranilast-NLRP3 were found to be 3.18 ± 0.006, 3.34 ± 0.062, 3.050 ± 004, 3.19 ± 0.005, 4.099 ± 0.008, 3.84 ± 0.006, and 4.35 ± 0.008 respectively. As minor fluctuations with less RMSD value indicate good system stability [53]. Overall the RMSD analysis indicates that the hits predicted as active against NLRP3 drug target from the ZINC, In-house and Phytochemical database, revealed low average RMSD values than reference compound (Table 4). The dynamic stability assessment of these compounds showed stable pharmacological behavior and may have better pharmacological efficiency in experimental set up.

**Table 4:** Average values of RMSD and RMSF of protein–ligand complexes

S/NO	Protein/ligand complex	Average RMSD	Average RMSF
1	ZINC12359085-NLRP3	3.18 ± 0.006	1.54 ± 0.05
2	ZINC72288245-NLRP3	3.34 ± 0.062	1.41 ± 0.04
3	BA-II-51-NLRP3	3.050 ± 004	1.19 ± 0.03
4	BA-II-45-NLRP3	3.19 ± 0.005	2.05 ± 0.06
5	5,280,448-NLRP3	4.099 ± 0.008	1.63 ± 0.04
6	115,089-NLRP3	3.84 ± 0.006	1.29 ± 0.02
7	Tranilast-NLRP3	4.35 ± 0.008	1.43 ± 0.06

### Residues flexibility indexing

**Fig. 4:**
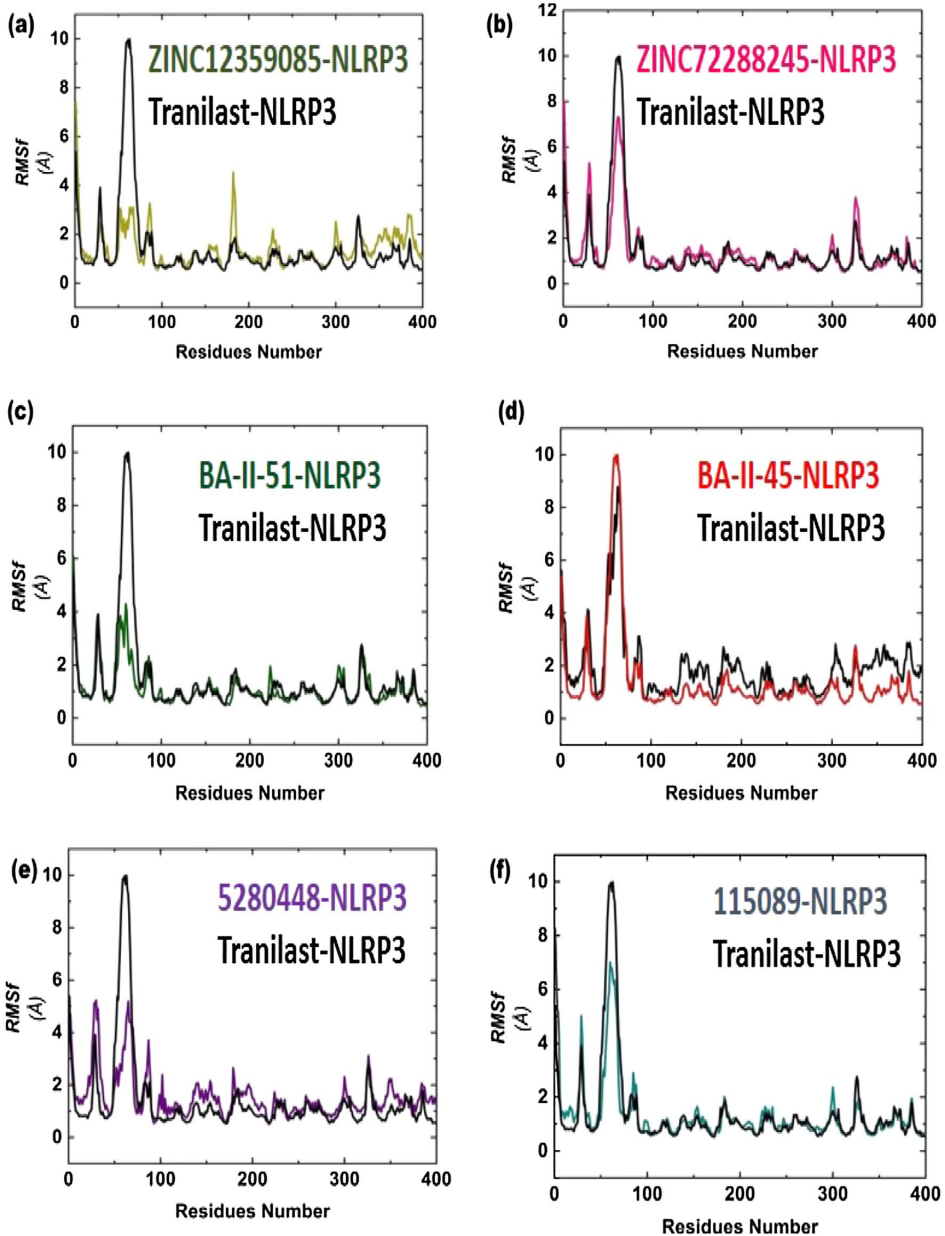
Black color indicates RMSF analysis for Tranilast-NLRP3 complex **a** ZINC12359085-NLRP3 complex **b** ZINC72288245-NLRP3 complex **c** BA-II-51-NLRP3 complex **d** BA-II-45-NLRP3 complex **e** 5,280,448-NLRP3 complex **f** 115,089-NLRP3 complex

Root mean square fluctuations (RMSF) measure the residues flexibility for each complex. The region with low RMSF lead to rigidity, while the high RMSF value indicate more flexibility. In case of ZINC database, ZINC12359085-NLRP3 complex showed 1–2 Å low fluctuations for residues 50–78 and 0.1–0.4 Å for residues 120–130, as compared with reference compound. However, some regions show local fluctuations (Fig. 4a). The ZINC72288245-NLRP3 complex revealed higher fluctuations at the region of 20–40 and 50–70 while the region between 100 and 300 presented local fluctuations by comparing with Tranilast-NLRP3 complex (Fig. 4b). Interestingly, for BA-II-51-NLRP3 complex low fluctuations in 50–70 residues (2–4 Å) and 250–300 (1–1.5 Å) were observed. The BA-II-51-NLRP3 complex acts as a better candidate as compared with Tranilast-NLRP3 complex (Fig. 4c). In case of BA-II-45-NLRP3 complex same fluctuation was observed as ZINC12359085-NLRP3 and BA-II-51-NLRP3 complexes (Fig. 4d). In case of phytochemical database, 5,280,448-NLRP3 and 115,089-NLRP3 complexes, similar RMSF and local fluctuations were observed (Fig. 4e and f).

In addition, the average RMSF value of all complexes, including ZINC1239085-NLRP3, ZINC72288245-NLRP3, BA-II-51-NLRP3, BA-II-45-NLRP3, 5,280,448-NLRP3, 115,089-NLRP3, and Tranilast-NLRP3 were found to be 1.54 ± 0.05, 1.41 ± 0.04, 1.19 ± 0.03, 2.05 ± 0.06, 1.63 ± 0.04, 1.29 ± 0.02, and 1.43 ± 0.06, respectively.

The average RMSF values for all the compounds ZINC72288245, BA-II-51, and 115,089 indicating potential interaction with NLRP3 protein (Table 4).

### Dynamic cross correlation matrix (DCCM)

**Fig. 5:**
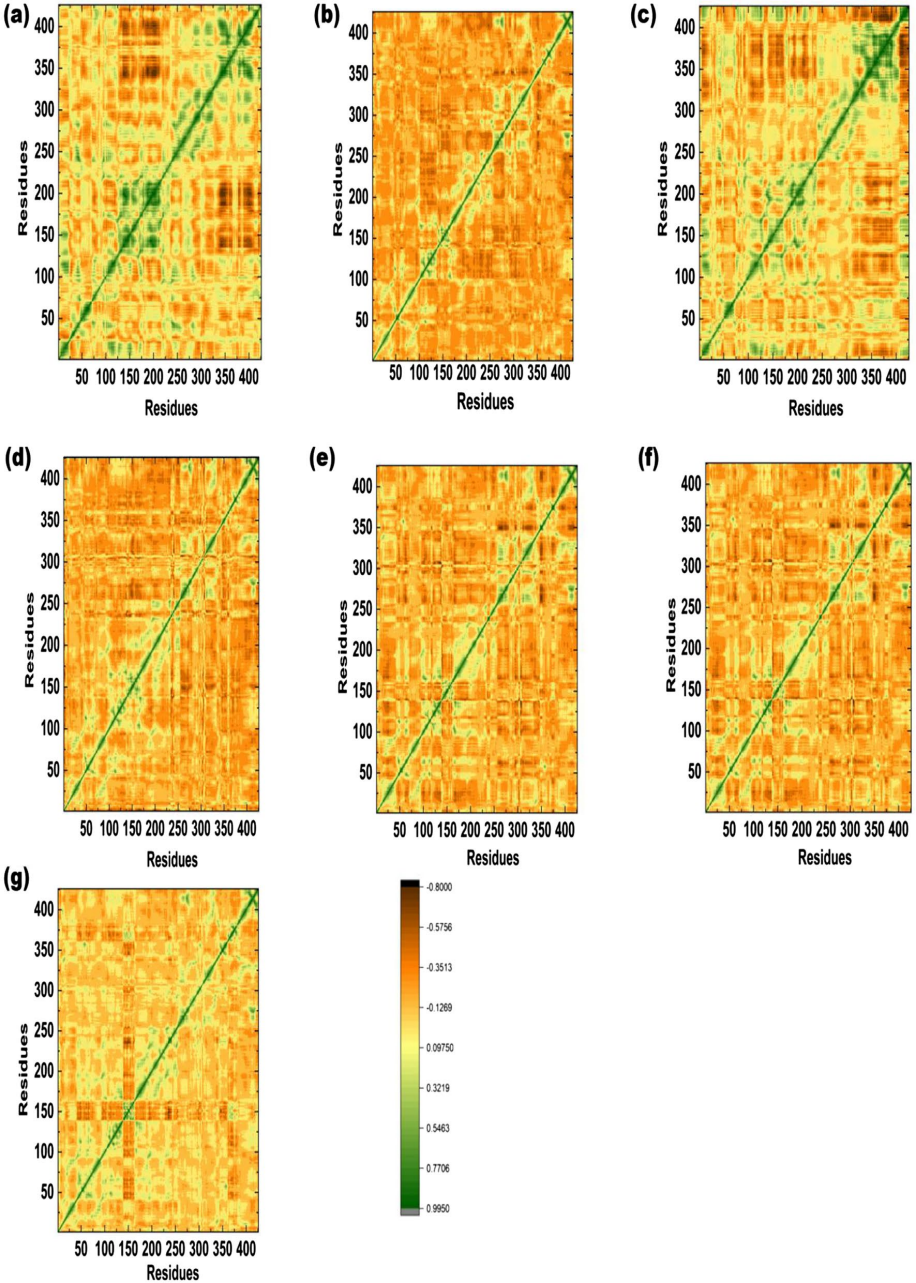
Presents the DCCM analysis **a** ZINC12359085-NLRP3 complex **b** ZINC72288245-NLRP3 complex **c** BA-II-51-NLRP3 complex **d** BA-II-45-NLRP3 **e** 5,280,448-NLRP3 complex **f** 115,089-NLRP3 complex **g** Tranilast-NLRP3 complex

DCCM was performed to understand the anti-correlated and correlated motion in NLRP3 and its docked complexes with ZINC12359085, ZINC72288245, BA-II-51, B-II-45, 5,280,448, 115,089. Seven plots for DCCM were generated, in which patterns of negative and positive correlated motions in protein–ligand complexes were presented in Fig. 5a–g. The deep green color indicates a positive correlation, whereas, brown color presents negative correlation. The positive correlated residues move in same direction and the negative correlated residues move in opposite direction. The DCCM analysis revealed that the binding site residues, where compound, BA-II-51 strongly bound, showed positive correlations as compared to reference compound (Fig. 5c). However, the BA-II-45, ZINC12359085, and ZINC72288245 in complex with NLRP3 showed significant positive correlation motion, with minor fluctuations were observed. In case of Phytochemical database, the compounds 5,280,448 and 115,089 in complex with NLRP3 protein, showed slightly weak negative correlation motion at 200–300 residues (Fig. 5e and f). The overall DCCM results showed that the selected compound might play significant role in the stability of these complexes.

### Clustering of protein’s motion

**Fig. 6:**
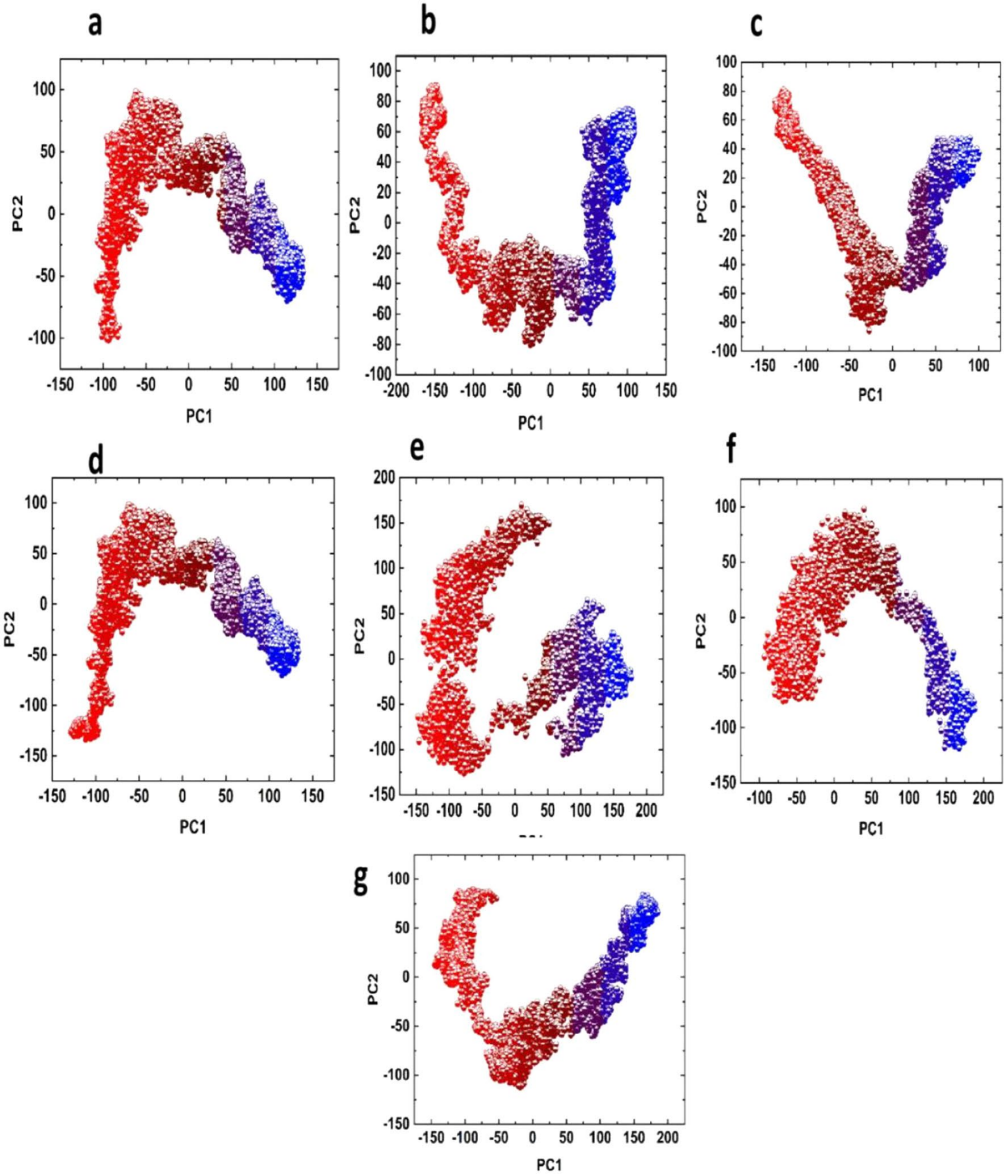
Principal component analysis for **a** ZINC12359085-NLRP3 complex **b** ZINC72288245-NLRP3 complex **c** BA-II-51-NLRP3 complex **d** BA-II-45-NLRP3 complex **e** 5,280,448-NLRP3 complex **f** 115,089-NLRP3 complex and **g** Tranilast-NLRP3 complex

Motion mode analysis was performed which explored the favorable conformational changes in the chemistry of bonded hits and target protein. The PCA analysis of top hits is given in Fig. 6. The ZINC12359805-NLRP3 and ZINC72288245-NLRP3 complexes consist of similar patterns of phase motion along with magnitude. The ZINC12359085-NLRP3 complex starts with red dot and end with blue dot, covering an area from − 100 to + 150 along PC1 and − 100 to + 100 along PC2 (Fig. 6a). The ZINC72288245-NLRP3 covering area of from − 175 to + 100 along PC1 and − 80 to + 80 along PC2 (Fig. 6b). The BA-II-51-NLRP3 complex was arranged and compact as compared with Tranilast-NLRP3 complex, beginning from red dot, ending to blue dot covering an area from − 120 to + 75 along PC1 and − 80 to + 80 along PC2. The Tranilast-NLRP3 complex was found to be so assembled and more dispersed, presenting that BA-II-51-NLRP3 complex showed good magnitude along with protein motion during 100 ns MD simulation period. The BA-II-51-NLRP3 complex residues are almost in same energy phase (Fig. 6c), showing stable behavior. As shown in (Fig. 6d), BA-II-45-NLRP3 complex, showed little disassembly but in arranged than Tranilast-NLRP3 complex, starting from red and ending in blue, covering space of − 75 to + 120 along PC1 and − 75 to + 90 along PC2. The PCA graph for 5,280,448-NLRP3 complex covering the space of − 150 to + 150 along PC1 and − 100 to + 130 along PC2 and 115,089 in complex with NLRP3 covering space of − 75 to + 150 along PC1 and − 75 to + 100 for PC2 (Fig. 6e and f). However, it was noted that Tranilast-NLRP3 complex was clustered and mixed (Fig. 6g).

Our results described that each compound showed different motions from other complexes. The change in motion of complexes indicated that binding of compounds induced less conformational impact on NLRP3 conformational dynamics, however, quite stabilization during MD simulation period.

### Hydrogen bond analysis

**Fig. 7:**
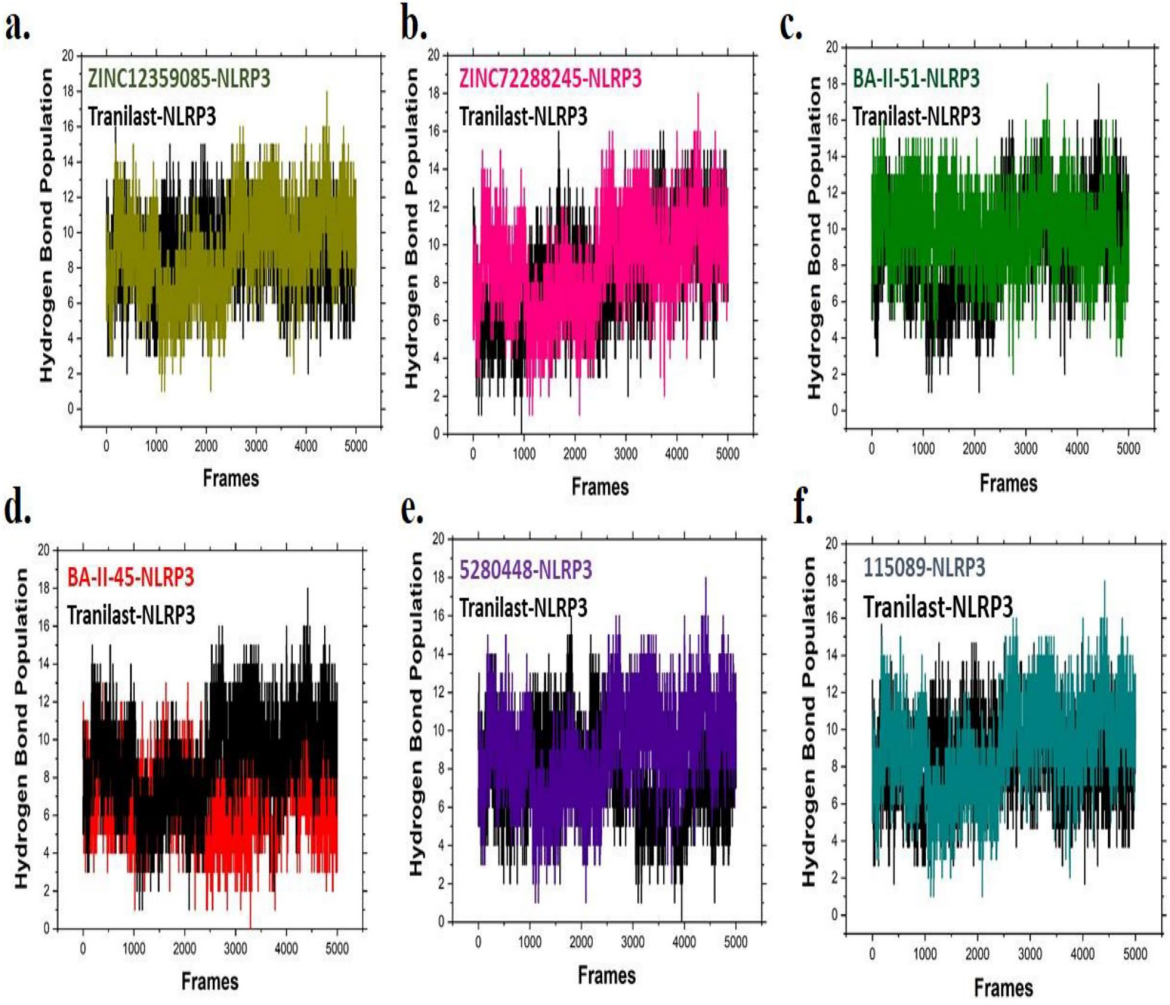
Presented the hydrogen bond analysis, where black color in all graphs represent hydrogen bond analysis for Tranilast-NLRP3 complex **a** ZINC12359085-NLRP3 complex **b** ZINC72288245-NLRP3 complex **c** BA-II-51-NLRP3 complex **d** BA-II-45-NLRP3 complex **e** 5,280,448-NLRP3 complex **f** 115,089-NLRP3 complex

Hydrogen bond analysis plays a significant role in observing the lifetime interactions between protein-ligands and helps to precise the atomic level analysis [54]. It’s possible to observe the hydrogen bond formation between NLRP3 and hits/reference at long production steps in MD in Fig. 7. The hydrogen bond analysis presented that both ZINC12359085 and ZINC72288245 compounds in complex with NLRP3 favored hydrogen bond formation as compared to Tranilast-NLRP3 complex (Fig. 7a and b). Binding of BA-II-51 with NLRP3 protein significantly increased the hydrogen bonding networks as comparison with Tranilast-NLRP3 complex (Fig. 7c). Interestingly, BA-II-45 has lesser effect in establishing hydrogen bonds (Fig. 7d). In case of 5,280,448 and 115,089 in complex with NLRP3 have less effect on hydrogen bond formation as compared with other compounds (Fig. 7e and f). Our hydrogen bond analysis indicated that most of our final compounds strongly bond with NLRP3, which may serve as a potent inhibitors.

### MMPBSA analysis

The binding free energy of top hits with NLRP3 protein were computed by using the MMPBSA approach. The binding free energy and its components, van der Waals energy, surface area energy, electrostatic contribution to the solvation free energy, and electrostatic energy are enlisted in Table 5. The binding free energy and its components calculated by MMPBSA approach are enlisted in Table 5**.** Our result showed that ZINC12359085-NLRP3 complex has the lowest binding free energy − 27.6039 kJ/mol, followed by ZINC72288245-NLRP3 complex, with value of − 25.4018 kJ/mol. The BA-II-51-NLRP3 complex obtained binding free energy of − 29.9161 kJ/mol, lower than the Tranilast-NLRP3 complex (− 25.0211 kJ/mol). Also, the BA-II-45-NLRP3 complex has a relative binding free energy of − 29.5539 kJ/mol. In Phytochemical database, the 5,280,448 and 115,089 compounds with NLRP3 obtained an affinity of − 22.7076 and − 25.4018 kJ/mol values, respectively. Overall results revealed that selected compounds have stronger binding energy than reference compound and might have a role in complex formation with NLRP3. It was also noted that relative binding free energy of all complexes were in agreement with RMSF, RMSD, PCA, hydrogen bond analysis, and DCCM analysis.

**Table 5:** The MMPBSA Binding Free Energy Calculation (kcal/mol) of selected hits

Compounds	VDW	EEL	ESURF	EGB	TOTAL
ZINC12359085-NLRP3	− 37.5939	− 23.4732	− 4.984	24.5632	− 27.6039
ZINC72288245-NLRP3	− 34.8148	− 17.9546	− 4.8781	32.2457	− 25.4018
BA-II-51-NLRP3	− 32.2448	− 24.7622	− 4.7821	31.8731	− 29.9161
BA-II-45-NLRP3	− 48.4418	− 217.2169	− 6.2746	242.3794	− 29.5539
5,280,448-NLRP3	− 35.5954	− 9.5877	− 4.9215	27.3970	− 22.7076
115,089-NLRP3	− 34.8148	− 17.9546	− 4.8781	32.2457	− 25.4018
Tranilast-NLRP3	− 38.4999	− 24.7469	− 5.1639	43.3896	− 25.0211

## Discussion

NLRP3 mediate caspase-1 activation and secrete cytokines IL-1β during microbial infection and cellular damage. However, its abnormal activation is accountable for diabetes, its complications, dementia and other inflammatory diseases [55–57]. A number of small molecule inhibitors for NLRP3 inflammasome have been identified, and some of them have demonstrated significant therapeutic potential. Some direct inhibitors are available for NLRP3 inflammasome including MCC950, CY-09, Tranilast, OLT1177, and Oridonin. However, to date, none of them have FDA approved [58]. As no FDA-approved drugs are available for NLRP3 inflammasome, so there is a need to develop safe and effective drugs for the NLRP3 inflammasome. The current study focuses on the development of new inhibitors for NLRP3 inflammasome.

Virtual screening is an essential computational approach, which is widely used in the process of drug discovery and development. The ligand-based pharmacophore model is commonly used to discover new and potent ligands or inhibitors by comparing their molecular similarity to known inhibitors for a specific drug target. This approach does not require information about the protein structure [59]. In previous study, we used pharmacophore based virtual screening for the identification of new inhibitors against STAT3 drug target [60]. In current study, we used pharmacophore based virtual screening for the identification of new inhibitors against NLRP3. Pharmacophore model was generated from reference compound and validated by GH score (0.76). The validated pharmacophore was used for the virtual screening of three databases. After pharmacophore based virtual screening, total number of 646 active hits were selected from these databases. During drug development process, it is important to analyze the pharmacological efficiency of a drug candidate. During drug-likeness analysis, total of 391 compounds were obtained from these databases and docked with NACHT domain of NLRP3. Based on docking score and binding interactions, the top six compounds, ZINC12359085, ZINC72288245, BA-II-51, BA-II-45, 5,280,448, and 115,089 were selected for MD simulation. During the docking simulation, it was observed that the interaction of six compounds exhibited good docking score and strong binding interactions compared to reference compound. In a recently reported study, molecular docking of berberine against NLRP3 was carried out [61]. The docking score of berberine was predicted as − 7.27 kcal/mol. The docking scores of our compounds ZINC12359085 (− 8.43), ZINC72288245 (− 9.89), BA-II-51 (− 12.65), BA-II-45 (− 10.98), 5,280,448 (7.98), and 115,089 (− 9.47) were good than berberine. The selected six compounds exhibited binding interaction with same active sites, as previously identified by Dekker et al. [62], Dos Santos et al. [36]. The critical residues of the NACHT domain consist of Ala227, Ala228, Arg351, Arg578, Glu629, and Thr662. By binding to these residues, it is possible to hinder the conformational change which is necessary for the initial stage of inflammasome activation [62]. By comparing this, the docking scores and binding interaction of six compounds were found to be superior compared to reference compound.

After docking, MD simulation was carried out for six compounds. These compounds represented good stability in MD simulation. The RMSD analysis indicated that the six diverse inhibitors, having better stability than reference compound. The RMSD analysis was further supported by RMSF, hydrogen bond analysis, and DCCM analysis, which revealed that all the six inhibitors revealed more stability as compared to reference compound. In addition, the MM/PBSA calculated from the last 500 frames found more negative ∆G binding value, than reference compound. Recent work, done by Patli et al. reported a similar pattern of RMSD and RMSF [63]. One of the active compounds predicted in this study with PubChem CID 5,280,448 (calycosin) is reported to be a primary active ingredient in Astragalus mungoholicus Bunge [64]. This compound possesses a number of pharmacological effects, such as anti-inflammatory [65, 66], anti-oxidant [67], and anti-osteoporotic [68] properties. The compound 5,280,448 (calycosin) can also be used to treat Osteoarthritis. It was reported that calycosin inhibits NF-κB activation and decreases the synthesis of pro-inflammatory cytokines, such as IL-1β, IL-6, and TNF-α. The active compound PubChem CID 115,089 (Enterodiol) was categorized as phytoestrogens because of its plant origin. Studies in the fields of epidemiology and pharmacology have demonstrated the protective properties of END, and in particular, its oxidation product enterolactone (ENL), against osteoporosis, cardiovascular disorders, hyperlipemia, breast, colon, prostate, and menopausal syndrome [69]. One of the identified active compounds ZINC12359085 contains carbonyl, amino functional groups, and thiadiazole ring. Thiadiazole is a five-membered scaffold, which is a common and crucial component of many drugs and it is reported that this compound may reveal antibacterial, antitubercular, analgesic, antiepileptic, antiviral, and anticancer properties [70]. The identified compound ZINC72288245 contains ethoxy, amide, sulphonyl functional group, and thiophen ring. Sulfonyl or sulfonamide functional groups are widely employed as pharmaceutical agents, and their uses in medicinal chemistry cannot be ignored [71]. Similarly, thiophene moiety is present in commercially available drugs, including Olanzapine, Benzocyclidine, Sertaconazole, Tioconazol, Dorzolamide, Tipepidine, and Tiquizium Bromide [72]. Overall, our study found that the newly identified inhibitors would be able to inhibit NLRP3 protein, which ultimately cures the disease.

## Conclusion

The current study aimed to design novel NLRP3 inhibitors by employing multi-level in-silico approaches. The molecular docking analysis resulted the top six compounds, demonstrated excellent binding interactions and docking score. The hydrogen and hydrophobic interactions between NLRP3 protein and these compounds revealed the involvement of key residues, namely, Ala227, Ala228, Arg351, Arg578, Glu629, and Thr662. They also exhibited drug likeness properties. MD simulations brought valuable insights into stability and behavior of six compounds, exhibiting notable affinity and stability than reference compound. These findings provide an excellent example of pharmacophore based virtual screening as practical approach to deign novel NLRP3 inhibitors against diabetes. The outlook of the current work is that some experimental approaches are require to confirm the activity of these compounds against NLRP3.

## Data Availability

All the data and its links are available in the manuscript.
